# Using self-determination theory to understand the social prescribing process: a qualitative study

**DOI:** 10.3399/BJGPO.2020.0153

**Published:** 2021-03-10

**Authors:** Sara Bhatti, Jennifer Rayner, Andrew D Pinto, Kate Mulligan, Donald C Cole

**Affiliations:** 1 Alliance for Healthier Communities, Toronto, Canada; 2 Centre for Studies in Family Medicine, Western University, London, Canada; 3 Dalla Lana School of Public Health, University of Toronto, Toronto, Canada; 4 Upstream Lab, MAP Centre for Urban Health Solutions, Li Ka Shing Knowledge Institute, Unity Health Toronto, Toronto, Canada; 5 Department of Family and Community Medicine, St. Michael’s Hospital, Toronto, Canada; 6 Department of Family and Community Medicine, Faculty of Medicine, University of Toronto, Toronto, Canada; 7 South East Grey Community Health Centre, Dundalk, Canada

**Keywords:** social prescribing, social determinants of health, primary health care, qualitative research, self-determination theory

## Abstract

**Background:**

Social prescribing (SP) assists patients to engage in social activities and connect to community supports as part of a holistic approach to primary care*.* *Rx: Community* was a SP project, which was implemented within 11 community health centres (CHCs) situated across Ontario, Canada.

**Aim:**

To explore how SP as a process facilitates positive outcomes for patients.

**Design & setting:**

Qualitative methods were used. Eighteen focus groups were conducted at CHCs or by video-conferencing, and involved 88 patients. In addition, eight in-depth telephone interviews were undertaken.

**Method:**

Interviews and focus groups were transcribed verbatim, and analysed thematically using a theoretical framework based on self-determination theory (SDT).

**Results:**

Participants who had received social prescriptions described SP as an empathetic process that respects their needs and interests. SP facilitated the patient’s voice in their care, helped patients to develop skills in addressing needs important to them, and fostered trusting relationships with staff and other participants. Patients reported their social support networks were expanded, and they had improved mental health and ability in self-management of chronic conditions. Patients who became involved in SP as voluntary 'health champions' reported this was a positive experience and they gained a sense of purpose by giving back to their communities in ways that felt meaningful for them.

**Conclusion:**

SP produced positive outcomes for patients, and it fits well within the community health centre model of primary care. Future research should examine the impact on health outcomes and examine the return on investment of developing and implementing SP programmes.

## How this fits in

SP has been identified as a way of addressing determinants of health in primary care settings. Existing literature has explored the implications of SP on patients’ health; however, the mechanism for how SP produces these outcomes has not been fully explored. SP facilitates the patient’s voice in their care, helps patients develop skills in addressing the needs that are important to them, fosters trusting relationships with staff and other participants, and had a tangible impact on patients' lives. These results will be useful for primary care providers interested in incorporating SP into their practice.

## Introduction

SP is a process of using a link worker to recommend patients engage in social activities and community supports in order to holistically address their needs.^1^ The prescription can take many forms, depending on the community and resources available, and patients' needs. It may include a knitting circle, walking group, bereavement support group, or volunteering. Multiple studies indicate that these prescriptions improve social inclusion,^2–5^ mental wellbeing,^6^ physical activity levels,^7^ and self-management of health.^8,9^ However, beyond identifying the impact of these prescriptions, it is equally important to understand how SP as a process facilitates these outcomes. As such, this article builds on the work done by Hanlon *et al*,^10^ Kellezi *et al*,^3^ and Payne *et al* ^11^ in trying to understand the 'how' of SP.

*Rx: Community* was undertaken in Ontario CHCs from 2018–2020 (described in depth elsewhere).^12^ The SP pathway implemented involved providers identifying a patient with unmet social needs (for example, a patient who is widowed frequently coming in for social visits) and jointly deciding what would work best for the patient. The provider would then refer the patient directly to the prescribed activity or to a SP navigator (that is, a link worker), if further discussions were needed. Afterwards, the patient would be supported in attending their prescribed activity or become involved as a health champion. The opportunity to become a health champion is a part of a SP model called collaborative practice in the UK and was not offered at all centres. It was unique in the sense that these individuals were not prescribed an activity to attend, but rather the opportunity to lead the development and creation of programmes within their centres as a volunteer.^13^ This study aimed to explore the impact on patients' health and wellbeing as they navigated this SP pathway, and to use SDT to understand how the pathway produced positive outcomes for patients within the CHC context.

## Method

### Study design

A qualitative case-study approach was chosen to understand patients' experiences.

### Theoretical framework

**Figure 1.**
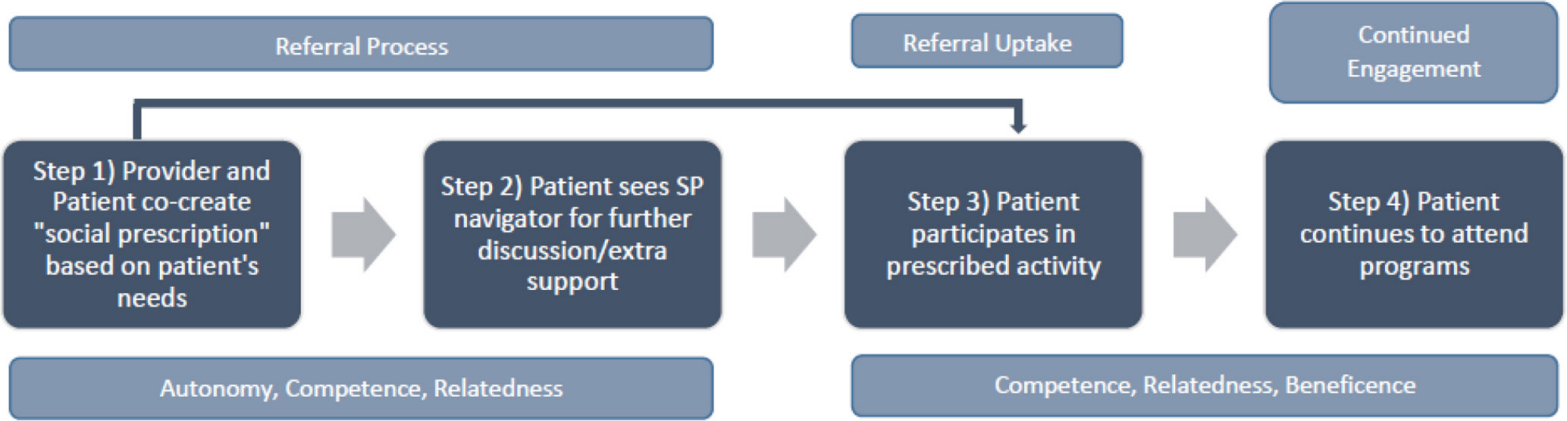
Theoretical framework of self-determination theory applied to the social prescribing pathway.

To study the SP pathway, a theoretical framework based on SDT, a theory of motivation, was applied (Figure 1).^14,15^ The theory suggests that those who experience self-determination (that is, the ability to make choices and exert control over one’s life) are more motivated to take action and experience greater psychological health and wellbeing. However, to become self-determined three basic psychological needs must be satisfied: autonomy (the need to feel control over one’s life and decisions); relatedness (the need to have close, affectionate relationships); and competence (the ability to influence outcomes, be capable, and effective).^14,15^ Beneficence (having a positive impact on others) has recently been identified as a fourth psychological need.^16^ SDT has been used as a theoretical framework to design and study interventions that sustain behaviour changes,^17–21^ and to study the impact of programmes, including SP.^10,22^

**Figure 2.**
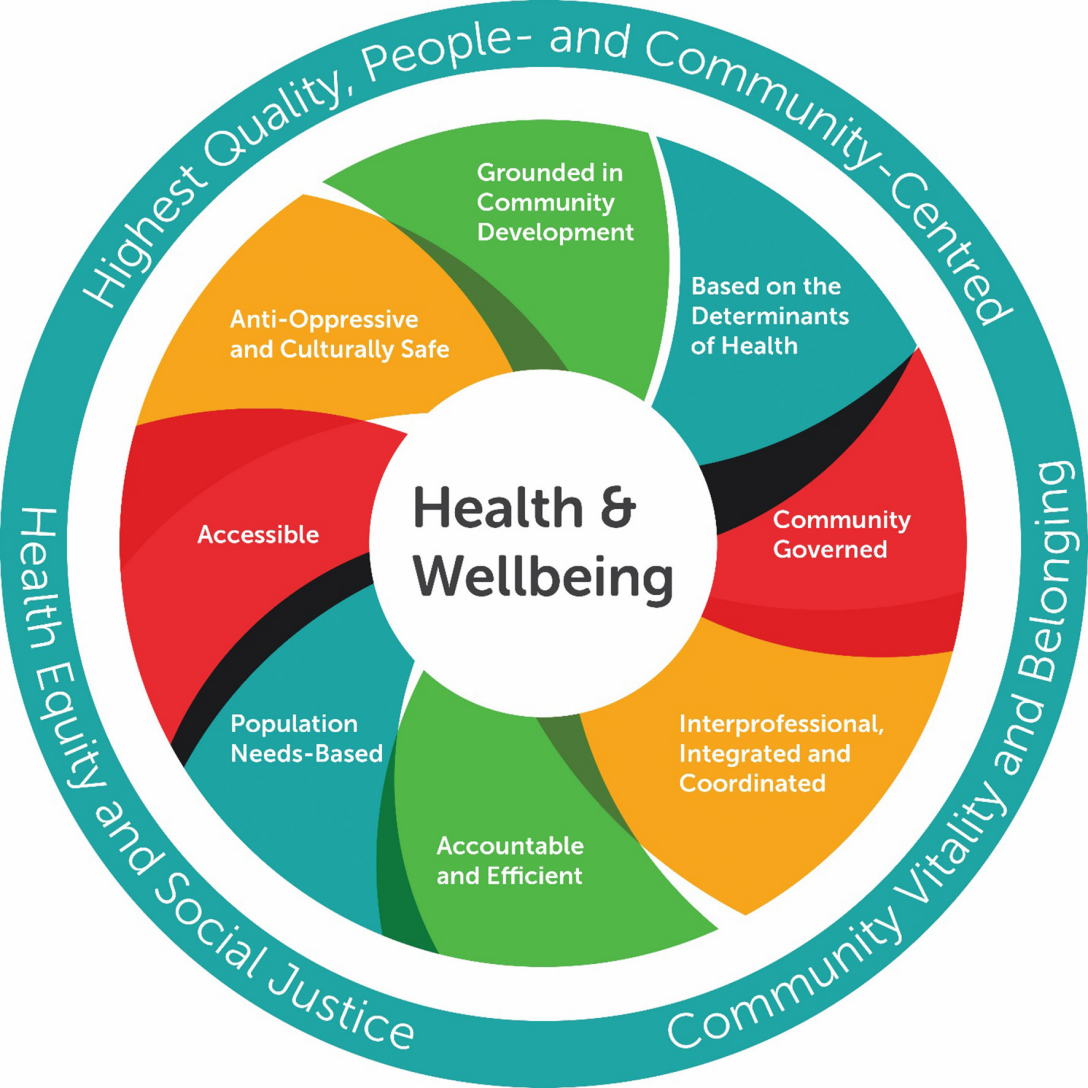
The Alliance for Healthier Communities' Model of Health and Wellbeing.

Self-determination at individual and community scales is a key component of health promotion theory.^23^ In this study, the SP pathway was implemented in the context of a comprehensive Model of Health and Wellbeing (see Figure 2).^12^ The model, used by all CHCs, is rooted in this understanding of health and wellbeing, health equity, health promotion, and community development.^24^

### Setting and participants

The study involved 11 Ontario CHCs including a mix of rural, urban, northern, and Francophone. Among those who had received a social prescription, convenience sampling was used to recruit participants through poster advertisements and staff contact. Those who agreed to participate were offered a free meal and reimbursement for transportation.

### Researcher characteristics

Research team members were of different sexes (three identified as women and two men), and of different cultural and disciplinary backgrounds. They included those with clinical experience working in primary care settings with diverse populations, substantial health promotion and population health experience, and varied methodological training, including qualitative and health services research. Three were staff at the Alliance for Healthier Communities, while two researchers were with other organisations; hence, bringing insider and outsider perspectives to the research team.

### Data collection

A semi-structured interview guide was developed to explore the referral process and uptake, and various outcomes (see Supplementary Appendix S1). From January 2019–October 2019, interviews and focus groups were conducted at approximately the 3-, 6-, and 12-month marks of the study. Focus groups conducted at the CHCs or through video-conferencing ran for 60–90 minutes. Interviews for those who could not attend a focus group or were uncomfortable in a group setting lasted from 30–40 minutes over the phone. Informed written consent was obtained from patients who participated. Interviews and focus groups were recorded and transcribed verbatim, with individual interview transcripts anonymised, and focus group transcripts only identifiable by the CHCs.

### Analysis

Qualitative analysis was conducted in two stages using NVivo (version 12). In the first stage, thematic analysis using an inductive approach was conducted on an initial set of transcripts to better understand the process and outcomes of SP as experienced by patients. The initial analysis prompted a theory to be chosen to help guide the second stage of analysis on how the SP pathway might facilitate these outcomes.

During the second stage, Braun and Clarke’s^25^ approach to thematic analysis was used. This involved reading through transcripts and generating initial codes using a deductive approach (for example, themes related to SDT, social isolation, loneliness, barriers, and so on) while still being open to new emergent themes and ones identified in the first round. Using the initial codes, one of the researchers and a research student used line-by-line coding to independently code transcripts. They then compared transcripts and generated a codebook, which was maintained by the researcher. By this time, it was apparent that enough interviews and focus groups had been conducted for no new themes to emerge. Transcripts were then coded for a second round and reviewed again to ensure credibility of analysis. Coders were in agreement for the majority of coding and disagreements regarding interpretation of codes were resolved between the coders. Codes were then collated into themes and subthemes and differences across CHC characteristics (that is, size, rurality, and age of CHC) were explored.

## Results

Ninety-six patients participated in this study. Eight individuals had one-on-one interviews and the remaining 88 took part in the focus groups. The majority of participants (see Table 1) were female and aged between 61 and 80 years, and of low income. More than half had secondary or equivalent education, lived with other people, and were from a White ethnic group.

**Table 1.** Patient participant characteristics (*N* = 96)

**Characteristics**	***n***	**%**
**Age, years**		
26–40	11	11.5
41–60	24	25.0
61–80	55	57.3
≥81	6	6.3
**Sex**		
Female	59	61.5
Male	29	30.2
Other (intersex, transgender, two spirit, other)	8	8.3
**Income**		
$0–$39 999	56	58.3
$40 000–$59 999	14	14.6
$60 000–$150 000	5	5.2
Other (do not know, prefer not to answer)	21	21.9
**Education**		
No formal education	2	2.1
Primary or equivalent	9	9.4
Secondary or equivalent	49	51.0
Post-secondary or equivalent	21	21.9
Other (do not know, prefer not to answer)	15	15.6
**Household composition**		
Multiple occupants	52	54.2
Single	28	29.2
Other (do not know, prefer not to answer)	16	16.7
**Ethnic group**		
White	63	65.6
Black	5	5.2
Asian	4	4.2
Indigenous	2	2.1
Latin American	3	3.1
Middle Eastern	1	1.0
Other (do not know, prefer not to answer)	18	18.8

Findings from patient interviews and focus groups were categorised into broad themes of context of care, processes of receiving a social prescription, and outcomes in their health and wellbeing (see Tables 2–4, with example quotes). Processes and outcomes related to elements of SDT are summarised in Tables 3 and 4.

**Table 2.** Context subthemes with examples (*n* = 18/29 transcripts)

**Subtheme**	**Examples**
Individualised care(*n* = 12/29 transcripts)	*'They always make you feel like--when you go there, especially the medical side, they make you feel like* […] *you have their undivided attention and they seem to be--you know, they’re concerned for your wellbeing. It makes you feel good when you go to them, because nothing worse than going to a doctor and feeling like, oh, you’re just a number …'* (Centretown patient)
CHC is a safe space(*n* = 6/29 transcripts)	*'Yes, another thing that I find for which I’m very grateful and surprised is how understanding people here are. It’s about one of the very few places that I feel welcome and respected as I am.'* (Centretown patient)

CHC = community health centre.

**Table 3.** Processes and relevance to self-determination theory components (*n* = 18/29 transcripts)

**Subtheme**	**Examples**	**SDT component(s)** **^a^**
Aligned with interests(*n* = 18/29 transcripts)	*'* *I’m diabetic, so type 2 diabetes and the stress eating one was referred to me by my doctor because she knew that I had at the time a very stressful call centre job, so--and you have a tendency to just eat while you’re stressed and I had an interest in controlling it.'* (Centretown patient) *'*[…] *I had been hesitant to volunteer about doing something specifically, committing--for a variety of my own personal reasons, so this idea of being able to give back in a way that I was comfortable with, that wasn’t dictated to me in any way, that could draw upon my strengths, my needs, and could be fairly flexible. That was one of the things I was looking for.'* (South Georgian Bay health champion)	A, C
Supportive staff(*n* = 18/29 transcripts)	*'I’m not a very trustful person, and I’m not a very open person, to be honest, even though it may seem it. They just--they seemed to be more--I don’t know, they just give you more a sense of comfort and that they’re not there to say, okay, well you should have done this, this and this, or you should do this, kind of more bossy or anything. They seemed more open to things and more willing to sit there and listen to you, and not--like, I’ve had doctors in the past where I’ve been in an abusive relationship and I’m afraid to say anything to them, where with these guys I’m more willing to sit there and tell them what’s going on, if there’s something wrong, and not worry that they’re going to judge me or force me to do something that I may not be ready to do at the time.'* (West Elgin patient) *'Yes, another thing that I find for which I’m very grateful and surprised is how understanding people here are. It’s about one of the very few places that I feel welcome and respected as I am.'* (Centretown patient)	R

^a^A = autonomy. C = competence. R = relatedness. SDT = self-determination theory.

**Table 4.** Outcomes and relevance to self-determination theory components (*n* = 25/29 transcripts)

**Subtheme**	**Examples**	**SDT component(s)** **^a^**
Social connections(*n* = 24/29 transcripts)	*'For myself, when I first started coming here, they gave me the opportunity to understand what the situation was, to understand what stress is, what type of stresses there were, and they gave me all these tools to how to help. But along with that, just within the groups I found that it’s not just attacking stress, it’s the friendships there that I’ve gained, the community, the laughter, everything attributes to this problem that they never mentioned in the course. These are things that also would help my stress.'* (Centretown patient)	C, R
Sense of community(*n* = 14/29 transcripts)	*'By giving of my time, I have felt much more integrated into the community … So by going out and doing things, I have felt--multiple things, not just this volunteer work, I feel like I’m getting back to being myself, to establishing what I used to be … and this is feeling like home.'* (South Georgian Bay health champion)	C, R
Improvement in self-management of health (*n* = 15/29 transcripts)	*'Sometimes you can feel so overwhelmed by the challenges you’re facing, but when you get involved with a group and with other people and you share, it can just be totally transforming of your life.'* (Centretown patient)	C, R
Improvement in mental health (*n* = 25/29 transcripts)	*'So this is kind of giving me a feeling of, you know what, you’re not useless. You don’t need a computer to be useful. There are other ways you can be useful and helpful, so that’s really important to me because it’s been many years for me now since I’ve really felt like, yeah, you know what, if I don’t have my part-time evening job, there’s something else that I can do.'* (Guelph health champion)	C
Positive impact on others(*n* = 12^b^/29 transcripts)	*'*[…] *it makes me feel good because I feel like I have helped other people, and that they are getting something from something that I’m doing. So it’s very valuable to the other people, and by them feeling good, it makes me feel like I’ve done something good.'* (Belleville and Quinte West health champion)	B

^a^B = beneficence. C = competence. R = relatedness. SDT = self-determination theory.

^b^100% of health champion transcripts mentioned beneficence.

### Context of care provided

#### Individualised care

Patients revealed key differences in care they received at their CHC. Outside of the CHC they reported having little to no voice in their care and/or treatment. They would simply receive a medical prescription and be on their way. In contrast, CHC staff would give their undivided attention, and work with them on trying to find a solution to the issues they faced.

#### CHC is a safe space

Patients described their centres as a safe space where they felt welcomed and not judged for talking about personal issues or life experiences. They credited staff for creating a space that accepted people from all walks of life.

### Processes of social prescribing

#### Aligned with interests

When facilitating social prescriptions, patients described providers respecting their needs and interests. They did not push their ideas or beliefs, but instead worked with them on finding the right solution. Patients appreciated providers framing prescriptions as 'is this something you would be interested in?' rather than 'this is what you should do'. For health champions, the ability to align their involvement with their passions (for example, music, gardening, and so on) and comfort levels, was the type of volunteering they were interested in, as they had decision-making power and weren’t simply filling a volunteer profile.

#### Supportive staff

Patients commented on how appointments felt more like personal interactions where you wouldn’t be patronised for doing the ‘wrong’ thing. Instead staff would listen to you, make you feel supported, and helped you find answers. Champions described the significance of staff brainstorming what can and cannot work instead of immediately shooting down their ideas and their ongoing support.

With respect to SDT, autonomy, competence, and relatedness were present in the broad theme of ‘processes’. They were operationalised by participants respectively as having decision-making power in regard to their care, addressing health needs that were important for them, and forming trusted and supportive relationships with their providers.

### Positive outcomes through engaging with social prescriptions

#### Social connections

For patients experiencing feelings of loneliness owing to their spouse or friends passing away, or arriving as a newcomer, attending their prescribed activity was their only opportunity to connect with others. They valued connecting with individuals with similar lived experiences (for example, traumatic brain injury, bereavement, and so on) as this helped them feel less alone. Health champions enjoyed volunteering because it allowed them to meet new people, brainstorm ideas as a group, and work together as a collective, which separated this work from traditional volunteering.

#### Sense of community

In addition to social prescriptions facilitating social connections, patients discussed how this led to developing a sense of community at the centre; for example, it was seen as a place of belonging where people cared for one another. One patient described how they now understood what the term community means; for example, she’s come to understand it as a place where you can connect with others, receive support, support others, and feel welcomed.

#### Improvement in self-management of health

Social prescriptions helped patients learn how to manage different aspects of their health, ranging from managing a new disability to living with anxiety and depression. The programmes helped patients understand what they were going through and helped them feel capable in taking care of their health. This skill was further developed for some patients through peer-to-peer learning when patients with similar lived experiences connected with one another. This created a safe space allowing each to share stories and, for many, hearing others successfully try a technique, encouraged them to try for themselves.

#### Improvement in mental health

Patients attended programmes because it improved their moods, helped them manage anxiety, or allowed them the opportunity to take a moment for themselves. For patients who had recently retired, or their children had moved out, they highlighted how these programmes kept them busy and gave them a reason to leave their homes. Champions discussed the importance of having the opportunity to feel useful after retiring or needing a break from full-time caregiving. Furthermore, being able to contribute to their community and seeing others enjoy their programmes gave them a renewed sense of purpose.

#### Positive impact on others

Champions enjoyed the opportunity to support others using their lived experiences and contribute to their communities in ways that felt meaningful for them. For example, a widowed champion started a bereavement support group called 'Life After Grief' to help others cope with loss, while another individual who used arts and crafts as a way to manage their anxiety, initiated a drop-in crafts programme. These individuals and many others spoke about how gifting their time not only benefited others but also themselves, and how this acted as a great motivator for them to continue volunteering.

Regarding participation in light of SDT theory, individual needs for competence, relatedness, and beneficence were satisfied. Respective examples were: learning how to manage their health, patients finding community, and having a positive effect on others.

## Discussion

### Summary

Using the theoretical framework, it was found that participants engaging in the SP pathway in a CHC setting, broadly satisfied the elements present in SDT. During the referral process (steps 1 and 2 in Figure 1), patients were supported in having a voice in their care (autonomy), they co-designed prescriptions based on their interests (competence), and created trusted relationships with staff (relatedness). When engaged with their prescriptions (steps 3 and 4 in Figure 1) patients were satisfying a need or developing a skill that was important to them (competence), creating meaningful relationships with other group participants, and developing a sense of community (relatedness). Although not present for all patients, the findings along with Hanlon *et al*’s^10^ suggest that components of SDT are reflected within the SP pathway, thereby making it a useful framework in understanding how SP as a process is impactful for patients.

The findings also suggest that along with prescribing programmes, it is equally important to help mobilise patients to create and deliver programmes themselves. In this study, health champions chose how they wanted to volunteer (autonomy), designed and led programmes based on their interests (competence), formed friendships with other champions (relatedness), and used their lived experiences to help others (beneficence). This opportunity proved to be a powerful enabler for patients' empowerment and the majority of centres involved transitioned into offering this opportunity as a result.

### Strengths and limitations

The use of focus groups as the main data collection method had both strengths and weaknesses. A group setting allowed participants to build off each other’s ideas; however, individuals who indicated agreement were not captured non-verbally, and individuals may have been less likely to voice disagreements, discuss personal barriers, and equally participate in discussions. The sample size was large; however, using a convenience sampling strategy potentially favoured patients with positive experiences and those who were physically able to participate, limiting the transferability of the results to other participants.

### Comparison with existing literature

While limited literature exists regarding how SP produces positive outcomes, the findings mirror themes identified in studies conducted by Hanlon *et al*,^10^ Kellezi *et al*,^3^ and Payne *et al*,^11^ among others that studied uptake and adherence of SP.^6,25–27^ Participants in Hanlon *et al*’s study emphasised the importance of having the power to set their own priorities in regard to their health.^10^ In all three studies, participants valued the personal and tailored interactions they had with their SP navigator and their use of an empathetic approach. The supportive nature of SP navigators made patients feel valued and listened to, and they appreciated their professionalism (for example, SP navigators following through with what they had been asked of by patients).^3,10,11^ Participants identified the social connections made with staff and other participants as contributing to their positive experiences. When individuals did not feel welcomed or integrated with the group they were more likely to cite the experience as negative.^3,10^ Individuals emphasised the value of shared experiences in helping them feel less alone^10^ and the importance of participating in these activities because it addressed a need important to them.^10,11^

In regard to the studies that explored uptake and adherence of prescriptions, autonomy was reflected in patients having a voice in their treatment and was deemed vital in increasing uptake of referrals.^7,26–28^ Competence, through aligning referrals with patients' interests, was found to increase the likelihood of patients identifying and following through with the referral.^27,28^ Regarding actual engagement with prescriptions, Husk *et al* ^28^ found that when participation resulted in a positive change (competence), adherence was more likely to be maintained. Relatedness could be described in other studies as using a person-centred care approach that was non-judgmental and facilitated a sense of trust.^7,26,27^

### Implications for research

This first Canadian SP study contributes to the existing literature on how SP as a pathway produces positive outcomes for patients, in light of a health promotion theory. By applying a theoretical framework, the study outlines how the concepts of autonomy, competence, relatedness, and beneficence are reflected within the key components of the SP process. This will be useful for future implementation and evaluation as they will have greater understanding on what makes a difference for patients and why. It is distinctive as it incorporated the involvement of health champions and was implemented in a team-based model of care within CHCs, with their focus on equity. Although this study has demonstrated SP to be a worthwhile intervention to become part of routine primary care, further investigation is required to understand what social prescriptions work best for whom, and how this could be implemented in different primary care settings in other countries.
